# P-501. Implementation of an Active Congenital Cytomegalovirus Screening Program in a Large Pediatric Hospital System. Key Findings and Lessons Learned

**DOI:** 10.1093/ofid/ofaf695.716

**Published:** 2026-01-11

**Authors:** Frank Esper, Lisa McBride, Patrick Burke, Carmen Jamis, Anirudha Das, Hany Aly, Samantha Anne, Colleen C Schelzig, Stephanie Jennings, Daniel D Rhoads, Hannah Wang

**Affiliations:** Cleveland Clinic Children's, Cleveland, OH; Cleveland CLinic CHildren's, Cleveland, Ohio; Cleveland Clinic, Cleveland, Ohio; Cleveland Clinic, Cleveland, Ohio; Cleveland CLinic CHildren's, Cleveland, Ohio; Cleveland CLinic CHildren's, Cleveland, Ohio; Cleveland CLinic CHildren's, Cleveland, Ohio; Cleveland Clinic Children's, Cleveland, OH; Cleveland CLinic CHildren's, Cleveland, Ohio; Cleveland Clinic, Cleveland, Ohio; Cleveland Clinic, Cleveland, Ohio

## Abstract

**Background:**

Congenital cytomegalovirus (cCMV) infection is the most common congenital infection. Cleveland Clinic (CC) recognized a substantial underdiagnosis of cCMV compared to the expected incidence. A multidisciplinary team at CC developed and implemented an active cCMV screening program for infants beginning Jan 2022 with a goal of improving detection and follow-up. This report highlights cohort outcomes and key challenges encountered during program execution.

**Figure:**
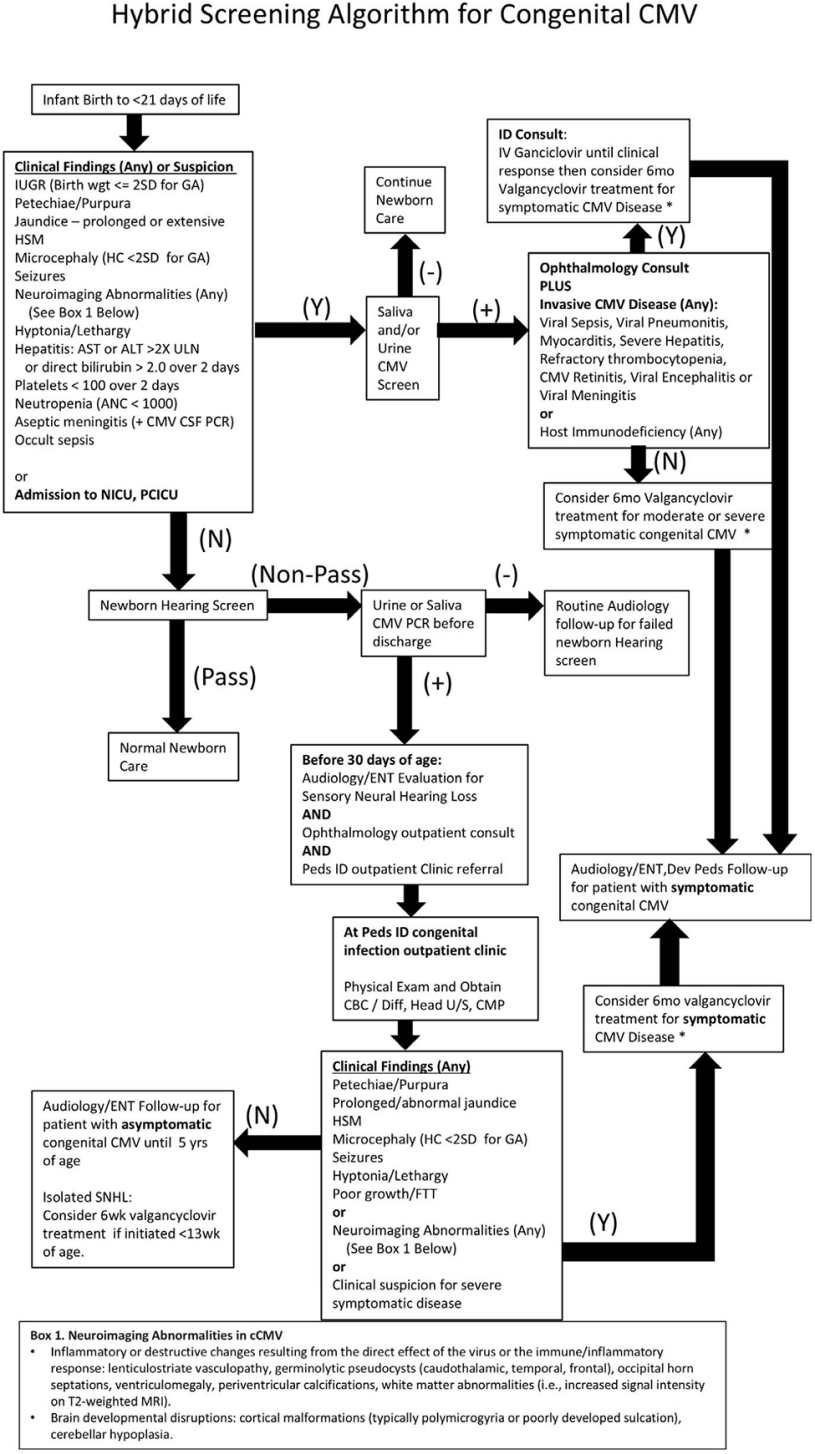
cCMV Screening algorithm Hybrid screening algorithm for congenital CMV (cCMV) at Cleveland Clinic: universal screening in NICU infants and targeted screening in newborn nurseries based on clinical signs or abnormal newborn hearing screen. Guides audiology follow-up and identifies candidates for antiviral therapy.

**Figure:**
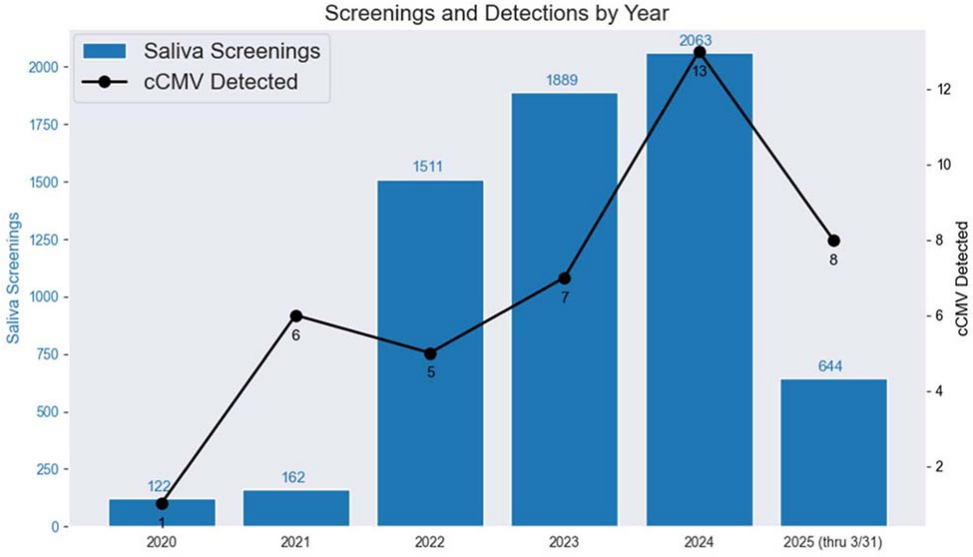
cCMV Screenings and Detections by Year Annual cCMV (congenital cytomegalovirus) screening volumes and corresponding detections are shown for the years 2020 through March 31, 2025. Both the number of screenings and the number of confirmed detections increased substantially over time. This analysis highlights the benefits of active newborn screening.

**Methods:**

With input from caregivers in Laboratory Medicine, Infectious Disease, Otolaryngology, Audiology, Newborn Nursery and Intensive Care, a standardized saliva screening program was developed and implemented. This protocol involves universal screening of newborns admitted to NICU and targeted screening in healthy newborn nurseries (clinical suspicion or abnormal newborn hearing screen (NHS)). Positive saliva screens were confirmed with urine PCR testing.

**Results:**

Over 12,000 deliveries occur annually across Ohio and Florida. From 1/1/2022 through 3/31/2025, there was a 1223% increase in screening compared to 2020-21. Of 5513 saliva screens that occurred across 16 newborn wards (9 NICU, 7 nursery) at 8 hospitals in 2 states, 918 were targeted and 4595 were universal. In total, 33 infants were identified by saliva screening and confirmed with urine testing (10 symptomatic with 2 deaths, 23 asymptomatic) nearly tripling the relative rate of identification. Of asymptomatic infants, 15 (65%) passed their NHS. Incidence of cCMV was 0.58% (1.42% targeted, 0.41% universal). The newborn nursery and ICU quickly adapted to using the program without a disruption in workflows. Substantial false-positive saliva results early in the program catalyzed changes in sample testing workflows and ultimately the testing platform. Infectious Disease and Audiology were instrumental in accommodating capacity for longitudinal follow-up and 1-3-6 benchmark auditory screening.

**Conclusion:**

Early multidisciplinary involvement and coordination are essential for successful implementation of cCMV screening. This program significantly increased the detection of cCMV in newborns despite many being asymptomatic with normal NHS, highlighting the importance of active screening programs.

**Disclosures:**

Samantha Anne, MD, Cochlear: Grant/Research Support|Eli Lilly: Advisor/Consultant|Plural publishing: royalties Daniel D. Rhoads, MD, Abbott: Grant/Research Support|Altona: Grant/Research Support|BD: Grant/Research Support|bioMerieux: Grant/Research Support|Cepheid: Grant/Research Support|Cleveland Diagnostics: Grant/Research Support|HelixBind: Grant/Research Support|Hologic: Grant/Research Support|Luminex/Diasorin: Grant/Research Support|Meridian: Grant/Research Support|Next Gen Diagnostics: Board Member|Next Gen Diagnostics: Ownership Interest|Pattern Bio: Grant/Research Support|Qiagen: Grant/Research Support|Q-Linea: Grant/Research Support|Rapid Diagnostics: Grant/Research Support|Renascent Diagnostics: Advisor/Consultant|Renascent Diagnostics: Financial payments|Renascent Diagnostics: Ownership Interest|Roche: Grant/Research Support|Selux Diagnostics: Grant/Research Support|Thermo Fisher: Grant/Research Support|Vela Diagnostics: Grant/Research Support Hannah Wang, MD, Cepheid: Grant/Research Support|Hologic: Advisor/Consultant|Hologic: Grant/Research Support|Moderna: Honoraria

